# The co-occurrence of sarcoidosis and anti-PLA2R-associated membranous nephropathy in a patient with underlying genetic susceptibility

**DOI:** 10.1186/s12882-024-03649-0

**Published:** 2024-06-27

**Authors:** Ying Ding, Yao Yao, Li Wan, Zhen Qu, Feng Yu

**Affiliations:** 1 Renal Division, Department of Medicine, Peking University International Hospital, Beijing, 102206 P.R. China; 2 Department of Pathology, Peking University Third Hospital, Beijing, 100191 P.R. China

**Keywords:** Sarcoidosis, Membranous nephropathy, PLA2R, HLA allele

## Abstract

**Background:**

Sarcoidosis is a multisystemic inflammatory disease, characterized by the presence of non-caseating, epithelioid granulomas. Glomerular disease in patients with sarcoidosis is rare and membranous nephropathy (MN) is cited as the most common. The association between the two diseases remained unclear. This article reported a case of co-occurrence of sarcoidosis and anti-PLA2R-associated MN, to provide a possible relationship between these two entities.

**Case presentation:**

A 61-year-old Chinese Han woman with a history of sarcoidosis was admitted to our hospital for nephrotic syndrome. Her sarcoidosis was diagnosed according to the adenopathy observed on the computed tomography scan and the biopsy of lymph nodes. The MN presented with nephrotic syndrome with a PLA2R antibody titer of 357RU/ml, and the final diagnosis was based on a renal biopsy. The patient’s sarcoidosis was remitted after treatment with prednisone. One year later MN was diagnosed, and she was treated with prednisone combined with calcineurin inhibitors, based on a full dose of renin-angiotensin system (RAS) inhibitor. The patient’s sarcoidosis had been in remission while the MN was recurrent, and her renal function deteriorated to end-stage renal disease 6 years later due to discontinuation of immunosuppression. A genetic test led to the identification of the HLA-DRB1*0301 and HLA-DRB1*150 genes associated with both sarcoidosis and MN, which provides a new possible explanation of the co-occurrence of these two diseases.

**Conclusion:**

This case suggested for the first time a potential genetic connection between idiopathic MN and sarcoidosis which needs further studies in the future.

**Supplementary Information:**

The online version contains supplementary material available at 10.1186/s12882-024-03649-0.

## Background

Sarcoidosis is a chronic multisystemic inflammatory disease of unknown etiology, characterized by the presence of non-caseating, epithelioid granulomas [1, 2]. The lung and lymphatic system are predominantly affected. Involvement of other systems may include skin, liver, spleen, brain, eyes, heart, kidney, bone, and joint. Formation of the granuloma is thought to be linked to genetic susceptibility and environmental exposures which trigger the interaction between CD4 + T cells and antigen-presenting cells [1, 3]. Although glomerular disease in patients with sarcoidosis is rare [4], among a broad spectrum of glomerular lesions that has been reported in sarcoidosis patients, membranous nephropathy (MN) is cited as the most common type [5]. Glomerular diseases might occur before, simultaneously with, or after the diagnosis of sarcoidosis [5].

MN is the most frequent cause of idiopathic nephrotic syndrome in adults [6]. The phospholipase A2 receptor (PLA2R) has been identified as the major antigen in primary MN since 2009 [7]. The detection of anti-PLA2R antibody had a specificity of 99% and sensitivity of 64% for the diagnosis of MN [8]. Noteworthy, anti-PLA2R antibodies could also be detected in secondary MN, such as lupus, HBV-associated, and cancer [9]. Furthermore, a high prevalence of anti-PLA2R-associated MN among patients with active sarcoidosis was also reported [10]. However, the underlying mechanism between sarcoidosis and MN remained unclear [5].

Herein, we report a case of coincidence of anti-PLA2R-associated MN and sarcoidosis, to provide a possible relationship between the two diseases.

## Case presentation

A 61-year-old Chinese woman was admitted for nephrotic syndrome. She presented with foamy urine and edema of the lower extremities for one month.

She had a history of hypertension for ten years, treated with nifedipine and irbesartan, with blood pressure controlled at 140/90mmHg.

**Fig. 1:**
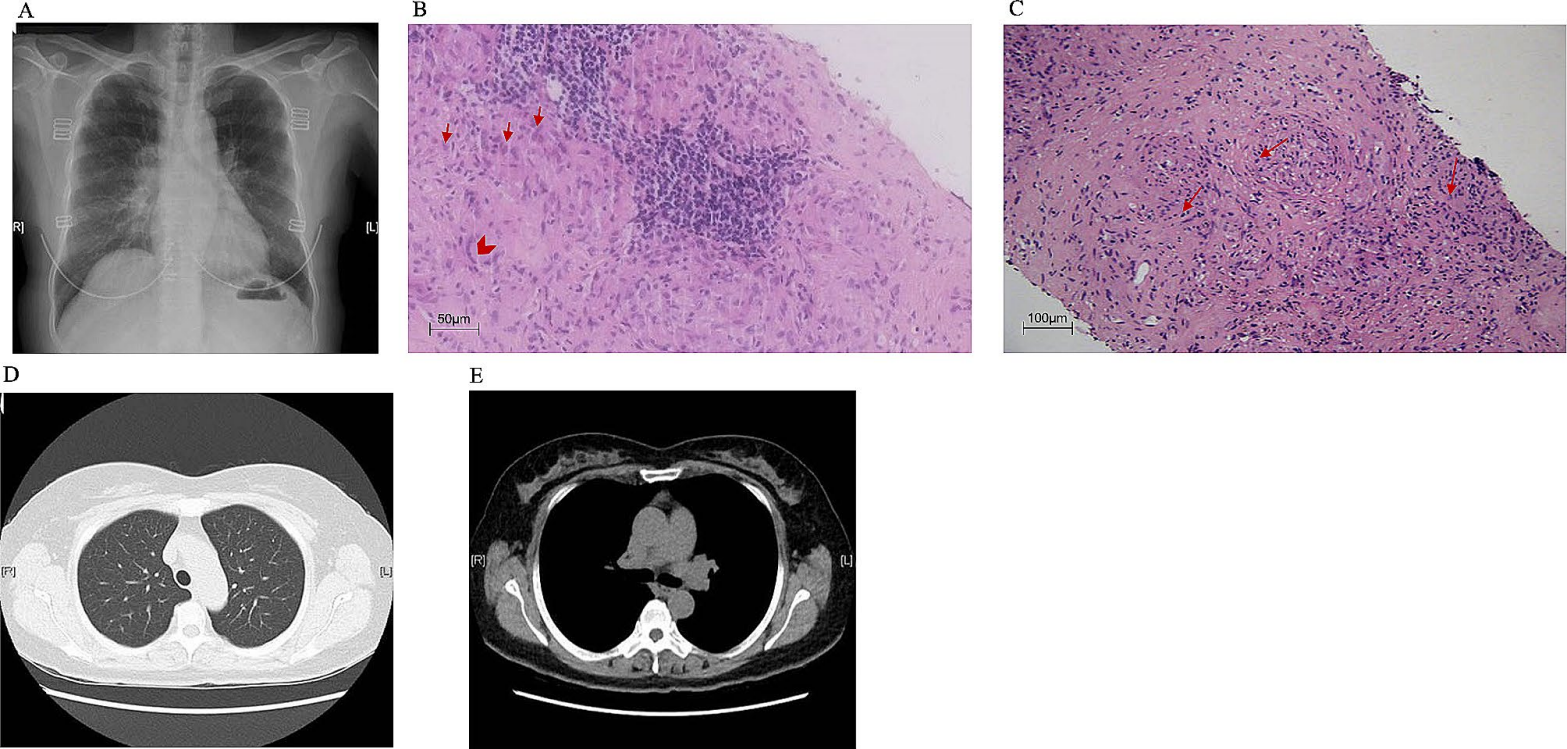
(**A**) The chest radiograph showed bilateral hilar adenopathy; (**B**) Biopsy of the mediastinal lymph node showed non-caseating granulomatous nodules consisting of epithelioid histiocytes (red arrow), multinucleated giant cells (red arrowhead), and a few lymphocytes (Hematoxylin and eosin staining; original magnification×40). (**C**) Biopsy of the right supraclavicular lymph node showed non-caseating granulomatous nodules consisting of epithelioid histiocytes (red arrow) and a few lymphocytes (Hematoxylin and eosin staining; original magnification×20); **D**, **E**: Computed tomography of the chest showed a resolution of the hilar adenopathy after one-year treatment by prednisone

She was diagnosed with pulmonary sarcoidosis with a complaint of chest distress and dry cough one year ago. Her serum calcium and angiotensin-converting enzyme (ACE) levels were normal. Purified protein derivative test and T-SPOT.TB assay [an interferon (IFN)-γ release assay which is based on detecting secreted IFN-γ in M. tuberculosis-specific T-cells stimulated by Mycobacterium-specific antigens] were all negative. The chest radiography showed mediastinal and bilateral hilar lymphadenopathy (Fig. 1A). Bronchoalveolar lavage showed lymphocytosis, with a CD4+:CD8 + ratio of 3.11. Biopsy of the mediastinal lymph node and right supraclavicular lymph node both showed non-caseating sarcoid granulomatous inflammation with negative acid-fast staining, confirming the diagnosis of sarcoidosis (Fig. 1B and C). At the time of diagnosis of lung sarcoidosis, no other organs were affected. Her urinalysis and serum creatinine were normal at that time. She had a good response to a moderate dose of prednisone at 0.45 mg/kg/d (30 mg/d), evidenced by the resolution of chest distress and hilar adenopathy (Fig. 1D and E). Prednisone was successfully tapered to 0.15 mg/kg/d (10 mg/d) within 6 months.

Besides, she used to work in an iron and steel research institution, with occupational metal dust exposure for twenty years. She had no family history of any autoimmune or kidney disease.

Physical examination on admission showed a temperature of 36.5 ℃,blood pressure of 134/80 mmHg, heart rate of 72 beats/min, and respiratory rate of 20 breaths/min, her body mass index was 26.3 kg/m^2^. A mild bilateral pitting edema was found in the lower extremities. No lymph node enlargement was found and chest auscultation was normal. The rest of the physical examination findings were also unremarkable.

**Fig. 2:**
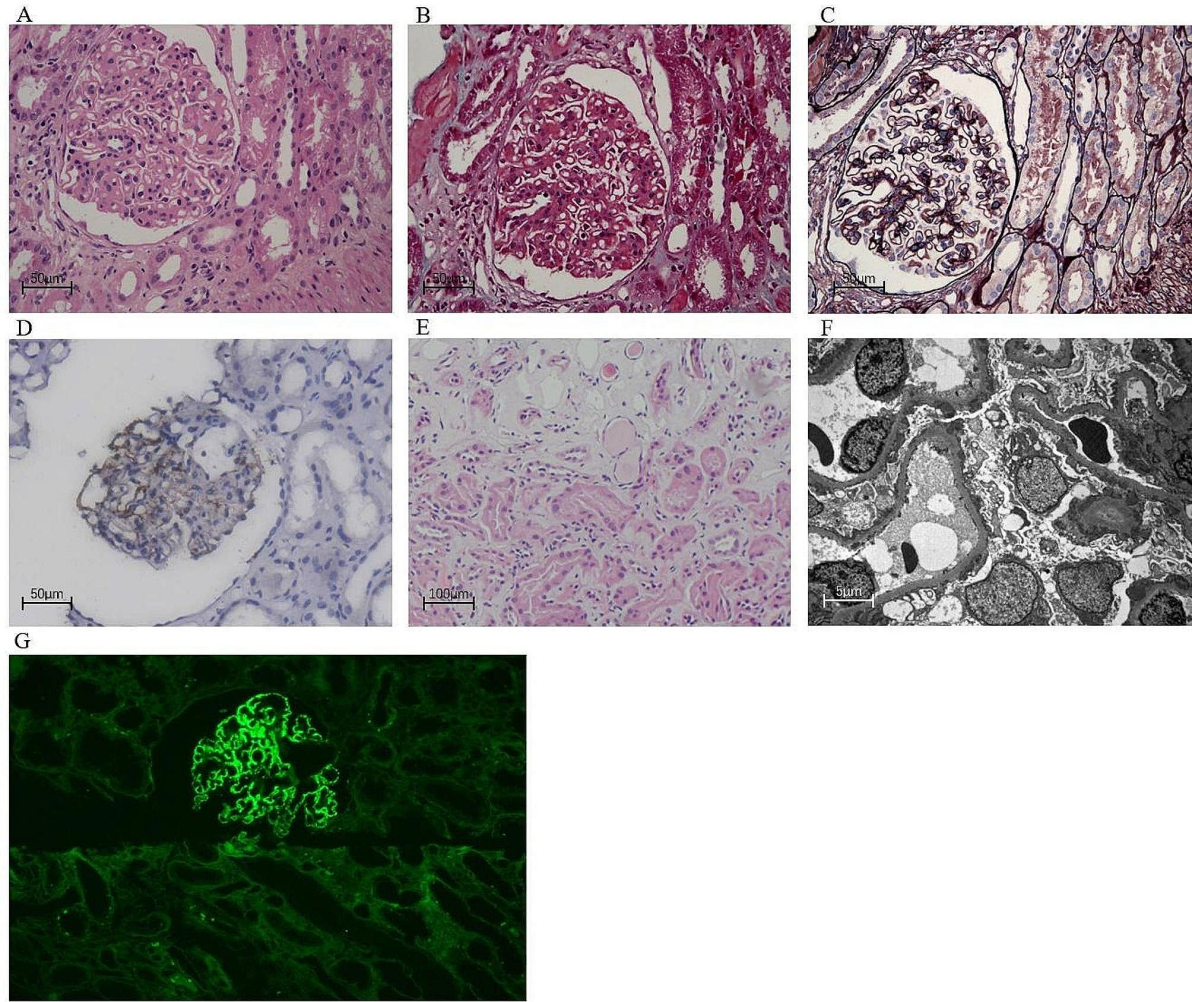
Histological analysis of the renal biopsy. (**A**) Light microscopy showed the thickening and stiffness of the capillary basement membrane (Hematoxylin and eosin staining×40). (**B**) Trichrome staining showed the presence of red subepithelial deposit (×40). (**C**) Periodic acid-silver methenamine staining demonstrated thickening capillary loops with vacuoles in some sections with spikes (×40). (**D**) Immunohistochemical staining showed PLA2R positivity (×40). (**E**) Small foci (about 10%) of lymphoid and mononuclear cell infiltration with fibrosis presented in the renal interstitium. Thickening of the walls of small arteries was noted (Hematoxylin and eosin staining×20). (**F**) Diffuse irregular thickening of the glomerular basement membrane, with high-density subepithelial electron-dense deposits and diffuse fusion of the podocyte foot process (4000X). (**G**) Glomerular paraffin fluorescence staining for IgG4(3+) (×20)

The urinalysis showed heavy proteinuria (+++) and mild hematuria (dysmorphic red blood cell at 5–10 /high power field). Urine protein excretion was 12 g/day. Her serum creatinine was 1.06 mg/dL, corresponding to an estimated glomerular filtration rate (eGFR) by CKD-EPI equation of 57 ml/min/1.73m^2^. Her serum albumin was 24 g/L, accompanied by dyslipidemia (low-density lipoprotein cholesterol was 6.89 mmol/L, triglycerides was 3.88 mmol/L). Her serum calcium was 2.38 mmol/L, which was also in the normal range. Complete blood cell count was normal, and erythrocyte sedimentation rate (ESR) was 23 mm/1 h (0–20 mm/1 h). The level of anti-M-type phospholipase A2 receptor antibody (anti-PLA2R Ab) was 357 RU/ml (reference range: 0-20RU/ml). HbA1c value was 5.7% (reference range 4–6%). The malignancies screening through serum tumor markers and imaging examinations were all negative. Serum protein electrophoresis disclosed no paraprotein and a normal free light chain ratio. Anti-nuclear antibodies, anti-neutrophil cytoplasmic antibodies (ANCA), and anti-glomerular basement membrane antibodies were negative. Hepatitis B and C, human immune-deficiency virus (HIV), and treponemal infection test were negative. Tests for serum and urine heavy metals overload (thallium, mercury, lead, and cadmium) were negative. No abnormality was found in thoracic computed tomography. Kidneys were of normal size, shape, and echogenicity under ultrasonographic examination. The patient subsequently underwent percutaneous renal biopsy. Renal biopsy showed typical MN. Immunofluorescence showed granular deposits of IgG (++++), C3 (++++), Kappa (+++) and Lambda (+++) along the glomerular capillary wall, negative for IgA, IgM, FRA, and C1q. Strongly positive for IgG4 was detected using Gene Tech Company’s mouse anti-human IgG Subtyping Reagents. Immunohistochemical staining showed PLA2R positivity (Fig. 2). The Electron microscope showed subepithelial electron-dense deposits, with some even throughout the basement membrane, along with podocyte foot process effacement diffusely (Fig. 2). She was diagnosed with anti-PLA2R-associated MN and sarcoidosis according to her previous history. Sarcoidosis was resolved at the moment, so treatment of MN was initiated with prednisone at 0.45 mg/kg/d (30 mg/d), cyclosporine (75 mg twice a day, with trough levels between 41 and 115ng/ml), angiotensin-II receptor blocker and anticoagulant.

Five months after renal biopsy, she had no remission of nephrotic syndrome with persistent heavy proteinuria (urine protein excretion between 12 and 20 g/24 h) and hypoalbuminemia (21–23 g/L). Her serum creatinine was between 0.98 and 1.30 mg/dl. Anti-PLA2R Ab level was 295 RU/ml. Her cyclosporine was not within the target levels and her creatinine was slightly elevated, so she discontinued cyclosporine and switched to daily oral tacrolimus at 0.045 mg/kg per day which was thought to be less renal toxic, with a trough level between 4.9 and 9 ng/ml. She continued taking prednisone at 30 mg/d. Rituximab was also recommended, but the patient refused due to economic reasons. To evaluate the disease activation of sarcoidosis: serum calcium, ACE level, ESR, and CRP were all in the normal range. Computed tomography of the thorax showed few effusions in the right thorax, but no evidence of adenopathy was found.

Fifteen months after the renal biopsy, her routine follow-up tests revealed 24-hour proteinuria of 6.16 g, serum albumin of 32 g/L, and serum creatinine of 1.06 mg/dl. The patient’s anti-PLA2R Ab level decreased to 52 RU/ml. No evidence of the complications of nephrotic syndrome, such as infection, embolism, or acute kidney injury, was noted. No evidence of sarcoidosis relapse was found on the computed tomography of the thorax, and her serum calcium and ESR were normal. With the tendency of elevation of serum albumin and decrease of anti-PLA2R Ab titer, the patient continued to receive prednisone and tacrolimus, accompanied by irbesartan 150 mg twice daily. Her blood pressure was well-controlled at 120–130/70–80 mmHg. Then her 24-hour proteinuria reduced to 3.32 g, serum albumin mounted to 38.5 g/l, and serum creatinine maintained at 1.23 mg/dl one month later. The entire course of the disease is summarized in Table 1.

**Table 1:** Laboratory indices and treatment of the patient

	Biopsy	3 months	5 months	9 months	12 months	15 months
Serum creatinine(mg/dl)	1.06	1.05	1.27	1.12	1.33	1.06
Serum albumin(g/L)	24	22	23	28	34.8	38.5
Urinalysis (Protein)	3+	3+	3+	3+	3+	2+
Proteinuria (g/24 h)	12	16.3	20	6.8	6.16	3.32
Anti-PLA2R Ab level (RU/ml)	357	/	295	/	/	52
Prednisone	30	30	30	20	15	12.5
Cyclosporine(mg)	75 Bid	75 Bid	/	/	/	/
Tacrolimus(mg)	/	/	1.5 Bid	1.5 Bid	1.5 Bid	1.5 Bid

Sadly, she discontinued all the immunosuppression and only took Chinese traditional herbs for more than one year, and finally, her renal function deteriorated to end-stage renal disease (ESRD) 6 years later with urine protein output of 3.55 g/24 h, serum albumin of 21.7 g/L, creatinine of 10.3 mg/dl (eGFR of 3.15 ml/min/1.73m^2^) and the PLA2R Ab titer rebounded to 632.59 RU/ml. Noteworthy, her sarcoidosis was still in remission at this moment.

As the patient had PLA2R-associated MN in the context of sarcoidosis, to explore the underlying mechanism between these two diseases, HLA (human leukocyte antigen) alleles genotyping at four-digit resolution was done. The patient was found to carry HLA-DRB1*0301 and HLA-DRB1*1501, two independent HLA risk alleles for both idiopathic MN [11] and sarcoidosis [12].

## Discussion and conclusion

MN is the major cause of nephrotic syndrome in adults. The etiology of approximately 80% of MN cases is idiopathic. Secondary causes include infection, malignancy, autoimmune disease, and drugs/toxins [6]. Anti-PLA2R Ab is the first diagnostic biomarker found in idiopathic MN (iMN), which could be detected in 70% of patients with iMN [7, 13]. Our patient was anti-PLA2R Ab positive, accompanied by renal pathology typical of MN, which strongly supports the diagnosis of PLA2R-associated MN. Nevertheless, secondary causes of MN should also be carefully considered, one of which is sarcoidosis [10].

Sarcoidosis preferentially involves the lung and lymph nodes but also affects other organs [1]. Renal involvement occurs in 10–20% of sarcoidosis cases and most often consists of disorders in calcium metabolism with or without nephrocalcinosis and nephrolithiasis, as well as granuloma formation within the renal interstitium [4]. Although rare, different glomerular diseases have been reported in patients with sarcoidosis, and the most frequent one was MN [4, 5].

The coincidence or causal relationship between MN and sarcoidosis remained unclear. By searching PubMed, 8 articles with full text regarding sarcoidosis and MN were noticed [5, 10, 14–20], which were summarized in Table 2. Among them, two articles mentioned the relationship between PLA2R and sarcoidosis. Knehtl et al. described for the first time PLA2R-associated MN in the context of sarcoidosis, suggesting a possible relationship between these two diseases [20]. Then, Stehlé T et al. retrospectively reviewed 9 patients with MN without evidence of secondary cause except for sarcoidosis. They found a high prevalence (5/9, 55%) of PLA2R antigens on renal biopsy. Interestingly, all five patients who were positive for PLA2R antigen had active sarcoidosis, and in the available follow-up sera of two patients, anti-PLA2R antibody followed sarcoidosis activity [10]. The above studies revealed that anti-PLA2R-associated MN might be secondary to sarcoidosis, but it is important to note that a causal relationship between the two disorders was found only in patients with active sarcoidosis at the onset of or during the course of MN. In our patient, sarcoidosis is the most suspicious secondary etiology. However, sarcoidosis-associated MN was not plausible in this patient as her sarcoidosis remained resolved at the onset and during the course of MN. Therefore, MN in our patient was inferred to be idiopathic rather than secondary to sarcoidosis.

**Table 2:** Reports describing the association of MN and sarcoidosis

Year	Reference	Age /Gender	Clinical/laboratory features	Renal pathology	Sequence of onset of sarcoidosis and MN	PLA2R Ab	Therapy	Outcome
1978	Mariani AF et al. ^(14)^	25/F 19/M	NS	MN	Sarcoidosis preceded the onset of MN by 6 months in the 25-year-old female patient and presented simultaneously with MN in the 19-year-old male patient.	NA	prednisone, CTX	resolution of proteinuria, stabilization of renal function
1979	Talyor TK et al. ^(15)^	57/M	NS, elevated Scr	MN with epithelial crescents	Sarcoidosis preceded the onset of MN by 10 years with progression of sarcoidosis.	NA	NA	NA
1989	Jones B et al. ^(16)^	32/F	proteinuria	MN	Sarcoidosis preceded the onset of MN by 5 years and recurred at the onset of MN.	NA	prednisone	resolution of proteinuria, relapse after ceasing prednisone
1994	Khan IH et al. ^(17)^	56/F	NS	MN and granulomatous interstitial nephritis	Simultaneously	NA	prednisone	decrease in proteinuria, stabilization of renal function
1999	Toda T et al. ^(18)^	49/F	Hypercalcemia, elevated Scr	MN and interstitial nephritis	Simultaneously	NA	prednisone	recovery of renal function and hypercalcemia
1999	Dimitriades C et al. ^(19)^	13/F	NS	MN	Sarcoidosis preceded the onset of MN by 1 year, and recurred at the onset of MN.	NA	prednisone, CTX	stabilization of sarcoidosis, resolution of proteinuria, normal renal function
2011	Knehtl M et al. ^(20)^	29/M	NS	MN and granulomatous tubulointerstitial nephritis	MN preceded the onset of sarcoidosis by 5 months.	seropositive for anti-PLA2R Ab and positive for PLA2R in renal biopsy	prednisone	resolution of nephrotic syndrome, stabilization of renal function
2013	Stehle T et al. ^(5,10)^	Total of 11 patients, 3 females and 8 males	seven of the 11 patients were NS	MN, 2 with GTIN	Three patients exhibited glomerular lesions simultaneously with sarcoidosis. MN preceded the onset of sarcoidosis in 5 patients (mean delay of 4.5 years). In 3 other patients, MN was diagnosed after sarcoidosis (mean delay of 12.6 years).	data available for 9 patients: 3 seropositive for anti-PLA2R Ab, 5 positive for PLA2R on renal biopsy	supportive care in 5 patients, prednisone in 6 patients	2 patients progressed to ESRD, and 1 patient died.

Note: CTX, cyclophosphamide; ESRD, end-stage renal disease; GTIN, granulomatous tubulointerstitial nephritis; NS, nephrotic syndrome; MN, membranous nephropathy; NA, not applicable; PLA2R, phospholipase A2 receptor

Even though MN in our patient might not be caused by sarcoidosis, these two rare autoimmune diseases that occurred in the same patient could not be that “coincident”. The underlying molecular link between sarcoidosis and MN remains unclear, as there was no specific antibody or target antigen has yet been identified [21]. The high prevalence of anti-PLA2R antibodies in MN associated with active sarcoidosis indicated that there might be some common predisposing factor in the pathogenesis process in these two diseases.

Interestingly, by searching the genetic background, genetic variations that could increase the susceptibility to both MN and sarcoidosis were found in our patient [1, 3, 6, 11]. HLA-DRB1*0301 and HLA-DRB1*1501 were both risk alleles identified for iMN in the Chinese Han population [11]. HLA-DRB1*0301 was found to be independently correlated with higher anti-PLA2R Ab levels, which plays a major role in MN disease occurrence and antibody production [22]. Besides, a study conducted by Wennerstrom A et al. observed that HLA-DRB1*1501 was a susceptible allele for sarcoidosis and HLA-DRB1*0301 was associated with resolving disease when compared with the persistent group [12]. The above indicated that MN and Sarcoidosis shared common susceptible alleles. In addition, a recent study showed that target antigens detected in sarcoidosis-associated MN reflect the overall incidence of target antigens in MN, in contrast to target antigens in other diseases associated with MN in which a distinctive target antigen has been identified [21], which indicated there exists differences in the relationship between sarcoidosis and MN from other secondary MN. The possible relationship could be a heightened immune response state induced by sarcoidosis in a background of genetic susceptibility which triggered the onset of MN [21]. Moreover, the response to treatment could also be explained by the alleles since HLA allele DRB1*0301, results in high levels of anti-PLA2R Ab and refractory MN [6] but is a favorable allele for good clinical response in sarcoidosis [12].

HLA risk alleles induced a disease-prone context in the patient, but the onset of the disease in a genetically susceptible person is usually triggered by additional insults. Previous studies demonstrated that air pollution and environmental exposures were positively associated with the prevalence of MN and sarcoidosis [23, 24]. The PLA2R mRNA was expressed in the kidney, lung, placenta, liver, and skeletal muscle [25]. It is speculated that environmental factors could damage the lung, and further trigger the production of anti-PLA2R Ab by upregulation of PLA2R expression in macrophage cells in pulmonary alveoli, and eventually develop autoantibody against PLA2R. After a thorough review of the previous history of our patient, a history of long-time metal dust exposure was noted which might contribute to the onset of her sarcoidosis and iMN.

Ultimately, our patients ended up with ESRD, the reason of which was multifactorial. First, this patient’s MN was at a high risk of progressive loss of kidney function due to her massive proteinuria and high level of anti-PLA2R Ab according to the 2021 KDIGO guideline [26]. Second, the patient carries the HLA allele which was independently correlated with higher anti-PLA2R Ab levels, contributing to the refractory to her MN treatment [22]. Third, the patient had been taking traditional Chinese herbs for more than one year, so drug-related kidney injury could not be excluded. Last but not least, the patient had poor adherence, was not followed regularly, and discontinued medication on her own, which participated greatly in her progression to ESKD. Renal ultrasound data at baseline and outcome showed the progression of the disease, with enhanced renal parenchymal echogenicity, which was presented in Supplementary Fig.  1.

In summary, we reported a patient who had both sarcoidosis and anti-PLA2R-associated MN and carried HLA-DRB1*1501 and HLA-DRB1*0301, the risk alleles for both diseases, which provides one more possible explanation for the association between these two diseases.

### Electronic supplementary material

Below is the link to the electronic supplementary material.

Supplementary Material 1: Supplementary figure: Figures A and B show the right and left kidneys respectively at the onset of the patient’s illness, and Figures C and D show the right and left kidneys respectively at the outcome.

## Data Availability

The data of this patient are available from the corresponding author upon request.

## Abbreviations

ACE: Angiotensin-converting enzyme
eGFR: estimated Glomerular filtration rate
ESR: Erythrocyte sedimentation rate
ESRD: End-stage renal disease
HBV: hepatitis B virus
HLA: human leukocyte antigen
MN: membranous nephropathy
PLA2R: phospholipase A2 receptor
RAS: renin-angiotensin system
