# Supplementary figures and images for: The co-occurrence of sarcoidosis and anti-PLA2R-associated membranous nephropathy in a patient with underlying genetic susceptibility
Source: BMC Nephrol. 2024 Jun 27;25:212. doi: 10.1186/s12882-024-03649-0 (PMC11212182; doi:10.1186/s12882-024-03649-0)

A

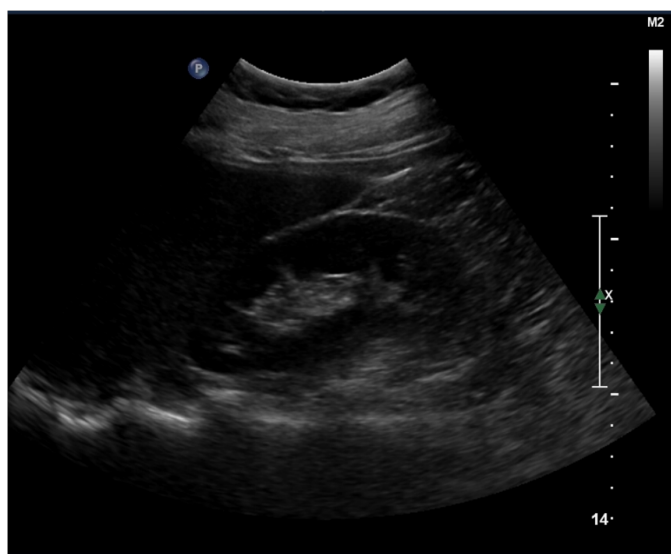

B

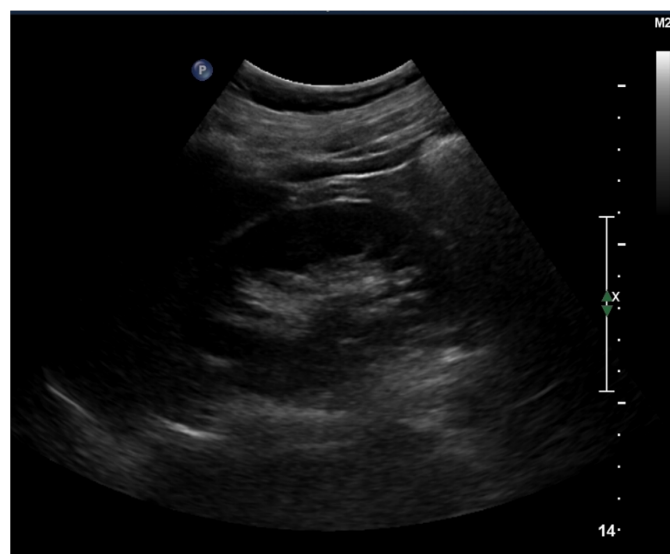

C

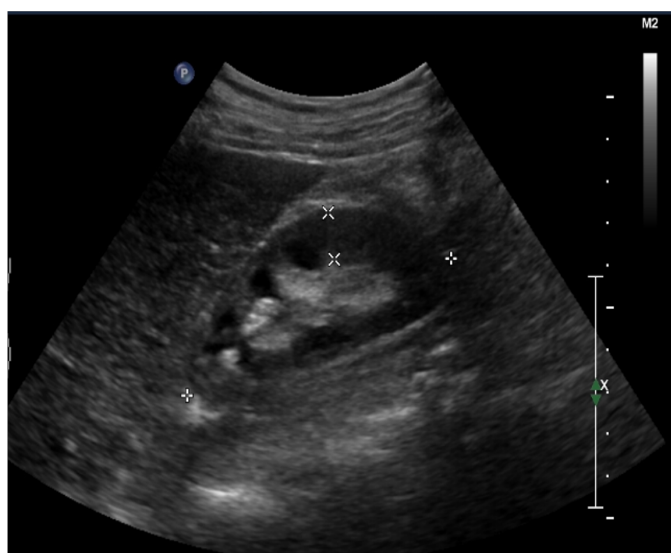

D

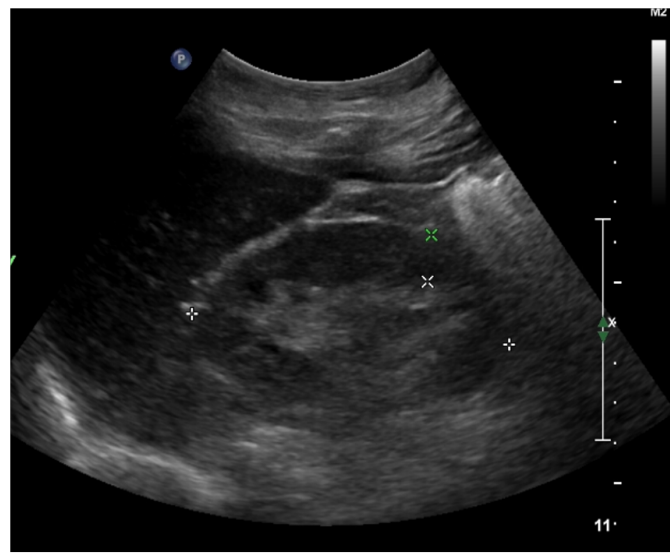

Supplement: Supplementary file 1 — Supplementary Material 1: Supplementary figure: Figures A and B show the right and left kidneys respectively at the onset of the patient’s illness, and Figures C and D show the right and left kidneys respectively at the outcome. [file 12882_2024_3649_MOESM1_ESM.pdf]
